# A *De Novo* Expression Profiling of *Anopheles funestus*, Malaria Vector in Africa, Using 454 Pyrosequencing

**DOI:** 10.1371/journal.pone.0017418

**Published:** 2011-02-25

**Authors:** Richard Gregory, Alistair C. Darby, Helen Irving, Mamadou B. Coulibaly, Margaret Hughes, Lizette L. Koekemoer, Maureen Coetzee, Hilary Ranson, Janet Hemingway, Neil Hall, Charles S. Wondji

**Affiliations:** 1 University of Liverpool, School of Biological Sciences, Cornwall House, United Kingdom; 2 Liverpool School of Tropical Medicine, Liverpool, United Kingdom; 3 Malaria Research and Training Center, Bamako, Mali; 4 Medical Entomology and Vector Control, Division of Virology and Communicable Disease Surveillance, University of the Witwatersrand and the National Institute for Communicable Diseases (NICD), Sandringham, Johannesburg, South Africa; Virginia Tech Virginia, United States of America

## Abstract

**Background:**

*Anopheles funestus* is one of the major malaria vectors in Africa and yet there are few genomic tools available for this species compared to *An. gambiae*. To start to close this knowledge gap, we sequenced the *An. funestus* transcriptome using cDNA libraries developed from a pyrethroid resistant laboratory strain and a pyrethroid susceptible field strain from Mali.

**Results:**

Using a pool of life stages (pupae, larvae, adults: females and males) for each strain, 454 sequencing generated 375,619 reads (average length of 182 bp). *De novo* assembly generated 18,103 contigs with average length of 253 bp. The average depth of coverage of these contigs was 8.3. In total 20.8% of all reads were novel when compared to reference databases. The sequencing of the field strain generated 204,758 reads compared to 170,861 from the insecticide resistant laboratory strain. The contigs most differentially represented in the resistant strain belong to the P450 gene family and cuticular genes which correlates with previous studies implicating both of these gene families in pyrethroid resistance. qPCR carried out on six contigs indicates that these ESTs could be suitable for gene expression studies such as microarray. 31,000 sites were estimated to contain Single Nucleotide Polymorphisms (SNPs) and analysis of SNPs from 20 contigs suggested that most of these SNPs are likely to be true SNPs. Gene conservation analysis confirmed the close phylogenetic relationship between *An. funestus* and *An. gambiae*.

**Conclusion:**

This study represents a significant advance for the genetics and genomics of *An. funestus* since it provides an extensive set of both Expressed Sequence Tags (ESTs) and SNPs which can be readily adopted for the design of new genomic tools such as microarray or SNP platforms.

## Introduction

The mosquito *Anopheles funestus* is a major vector of malaria throughout much of sub-Saharan Africa. Its efficiency as a vector is partially conferred by its highly anthropophilic and endophilic behaviours and in many places, parasite infection rates of *An. funestus* exceed those of *An. gambiae*, the other major malaria vector [1]. However, it has only recently started to receive the scientific attention such an important public health pest deserves. This is in part due to its relatively intractability to work with and difficulties in establishing laboratory colonies. Hence, there are far less genetic tools available for *An. funestus* than for *An. gambiae*. For example only 75 microsatellite loci have been identified in *An. funestus* compared with 300 in *An. gambiae* ; 600 Single Nucleotide Polymorphisms (SNPs) have been reported for *An. funestus* [2] compared to 400,000 in *An. gambiae* and only 2,846 ESTs (Expressed Sequence Tags) have been reported in *An. funestus* [3] compared to 153,165 in *An. gambiae* (http://www.ncbi.nlm.nih.gov). The genome of *An. gambiae* was sequenced in 2002 [4] while the genome of *An. funestus* is yet to be sequenced. This lack of genetic information has also resulted in a paucity of genetics studies in *An. funestus*, limiting the understanding of the biology of this important and neglected malaria vector. As an example, the availability of the high-resolution genetic information in *An. gambiae* has enabled microarray studies to be carried out in *An. gambiae* [5], [6] in order to elucidate various aspects of the its biology. No such microarrays have been designed for *An. funestus*. There is therefore an urgent need to design more genetic tools for *An. funestus*.

Transcriptome sequencing is a suitable alternative to whole genome sequencing, because it is significantly cheaper, but still provides information for the transcribed portions of genes. Indeed, next-generation 454 sequencing allows highly parallel DNA sequencing that is many orders of magnitude faster than standard sequencing methods and increase sequencing depth and coverage while reducing time, labour and cost [7]. Additionally these sequencing methods allow *de novo* sequencing, assembly and annotation of expressed genes [8] which makes it very suitable for a non-model species such as *An. funestus*. This will enable functional genomic studies in *An. funestus* using gene expression analysis, genetic and association mapping using the abundant SNPs identified.

454 sequencing could also allow differential gene expression analysis of the whole transcriptome between different phenotypes such as insecticide resistant and susceptible mosquitoes.

Here we report the comparative cDNA sequencing, using the 454 platform, between a pyrethroid resistant laboratory strain of *An. funestus* and a susceptible field sample. The main aim of this study was to generate functional genomic tools for this species. Additionally, we also attempted to identify potential genes associated with pyrethroid resistance by comparing the expression profiles of the two strains although different mosquito life stages where pooled for the sequencing.

## Results and Discussion

### Raw 454 data and assembly

**Figure 1:**
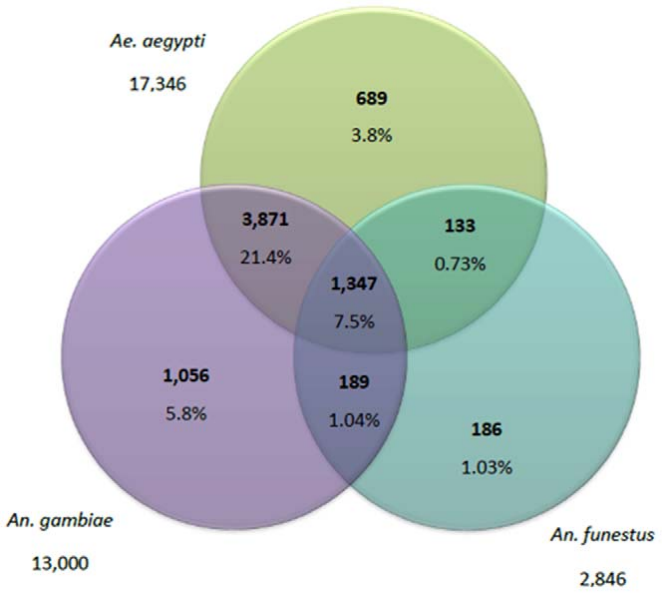
Venn diagram showing distribution of similarity search results. Numbers are the sum of unique contigs matching *An. gambiae*, *Ae. aegypti* and *An. funestus* and given with their relative percentage.

The Roche 454 FLX platform produced 375,619 reads totalling 68,308,429 bp with an average read length of 182 bp similar to the 197 bp observed for the butterfly *Meliteae cinxia* [8]. One quarter of the reads was between 237 bp and 277 bp in length and 50% of the reads between 185 to 285 bp in length. This dataset was trimmed using in-house tools to 213,410 reads of >30 bp in length and totaling 40,078,792 bp with an average read length of 188 bp. The reads were assembled with Mira 2.9.15 [9] to create 18,103 contigs (average length of 253 bp) using 149,406 reads, of which 1,039 contigs were at least 500 bp in length, similar to that obtained by 454 pyrosequencing in other insects such as *M. cinxia* [8] and the six-spot burnet moth, *Zygaena filipendulae* [10]. The average depth of coverage of these contigs (number of reads assembled into a contig) was 8.3 similar to that of *M. cinxia* [8]. The sequencing reads have been deposited to NCBI's SRA database with the assembly output (Accession number: SRA009034) and contig sequences have been submitted to the Transcriptome Shotgun Assembly sequence database (TSA) of NCBI (accession number: EZ915182 - EZ933284). A comparison of the 18,103 *An. funestus* 454 contigs to *An. gambiae* and *Aedes aegypti* total transcript sets from Vectorbase (with TBLASTX, E = 10^−3^) as well as previously published *An. funestus* ESTs from Genbank showed a large degree of overlap between these species (Figure 1). 41.2% (7,471) of the 18,103 contigs match an EST in these three transcriptomes. 65% (1855 ESTs) of the 2,846 already published *An. funestus* ESTs (using SANGER sequencing) was represented in this 454 transcriptome. As expected, this percentage was lower for *An. gambiae* (49.7%) and *Ae. aegypti* (32.2%). The percentage of annotated *An. funestus* ESTs (41.2%) obtained in this analysis is similar to that observed in other large assembled EST data sets of non-mammalian species suggesting that the un-annotated contigs and even the singletons could represent a substantial portion of *An. funestus* transcriptome [8], [10].

The assembly used 149,406 of the 213,410 reads, making use of 28,853,866 bp (72% of trimmed sequence). The remaining reads were singletons or considered either erroneous or contaminations. To test for these conditions, the unused reads were compared to nr database (the largest nucleotide database available through NCBI BLAST). Of the 64,004 unused trimmed reads, 58,205 were at least 50 bases in length and therefore suitable for BLAST with default parameters. 13,716 (23.5%) match a reference sequence in nr (BLASTX, E = 10^−5^, or 17,815 matches for E = 1) suggesting that a proportion of these singletons represents genuine ESTs but with probably low level of expression.

The 375,619 reads consisted of two independent 454 runs each covering half a plate. One run was from an insecticide resistant laboratory strain (FUMOZ) and the other from an insecticide susceptible field strain (Kela). The resistant run produced 170,861 raw reads, reducing to 98,021 after trimming and counting only those over 40 bp. The susceptible run produced 204,758 raw reads, reducing to 107,714. Assembly used 73,589 and 75,817 of these reads for resistant and susceptible strains respectively. Of the unused reads which 11,740 match nr, 6,574 were resistant reads and 5,166 were susceptible (BLASTX, E = 1) (Table 1).

**Table 1:** Summary of reads number between samples.

	Raw reads	Trimmed reads	Reads used in Assembly	Unassembled reads, with annotation
**Resistant (FUMOZ-R)**	170,861	100,521	73,589	6,092
**Susceptible (Kela)**	204,758	109,733	75,817	8,505
**Total**	375,619	210,254	149,406	14,597

### Assembly quality

To assess the quality of the assembly, the 18,103 contigs were compared to themselves and to the unused reads of over 50 bp. A good assembly of the 454 reads should normally produce contigs with minimal sequence similarity (apart from conserved regions) to other 454 contigs. A successful comparison required the subject to be a full match to any part of the query. 6,269 contigs showed variable degree of similarity with other contigs which is higher than that observed in a similar analysis for *M. cinxia* where only 8% of contigs had a match another contigs [8]. Comparing the unassembled reads to the contigs, 12,775 reads over 50 bp in length matched the contigs. Considering the 6,269 contig-to-contig matches, the average redundant contig length is 151 bp, this ranged from 40 to 285 bp. The average target contig is 206 bp, this ranged from 40 to 2,126 bp. Considering the 12,775 read-to-contig matches, the average improperly unused read has a length of 171 bp, ranging from 50 (the minimum cut-off) to 285 bp. The average target contig is 216 bp, ranging from 50 to 2,126 bp.

The 6,269 contigs that match a query (i.e. exactly or partially match another contig) can only be assembly error, presumably cases where initially different contigs are built up using different reads with the same sequence, leading to the contigs independently converging to the same sequence. Contig diversity varies considerably, 6,269 of the 18,103 contigs share some similarity with other contigs when tested with E = 1. Consequently, the assembly demonstrates both error and sequence conservation in gene families, with 6,269 contigs showing some similarity and 11,834 unique contigs. These matches between contigs could also be due to conserved motifs in different genes, or the presence of variants of single genes created by alternative splicing or allelic differences within the population of individuals sequenced [8].

The failure of 12,775 reads to join a matching contig can be put down to basic assembler error or low quality sequences [7]. 454 sequencing produces a quality score for each base of sequence, which can be used by the assembler to compare likely reliability of key reads. Quality comparison of the assembled reads, unused reads and improperly unused reads shows the quality of the improperly unused reads was higher than other reads.

**Figure 2:**
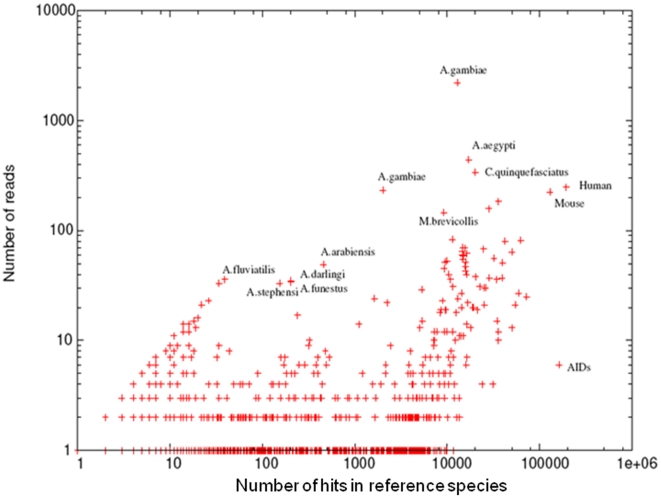
Number of hits in reference species in the nr database using BLASTX (E = 1) of the unassembled reads. Corresponding species have been labelled.

**Figure 3:**
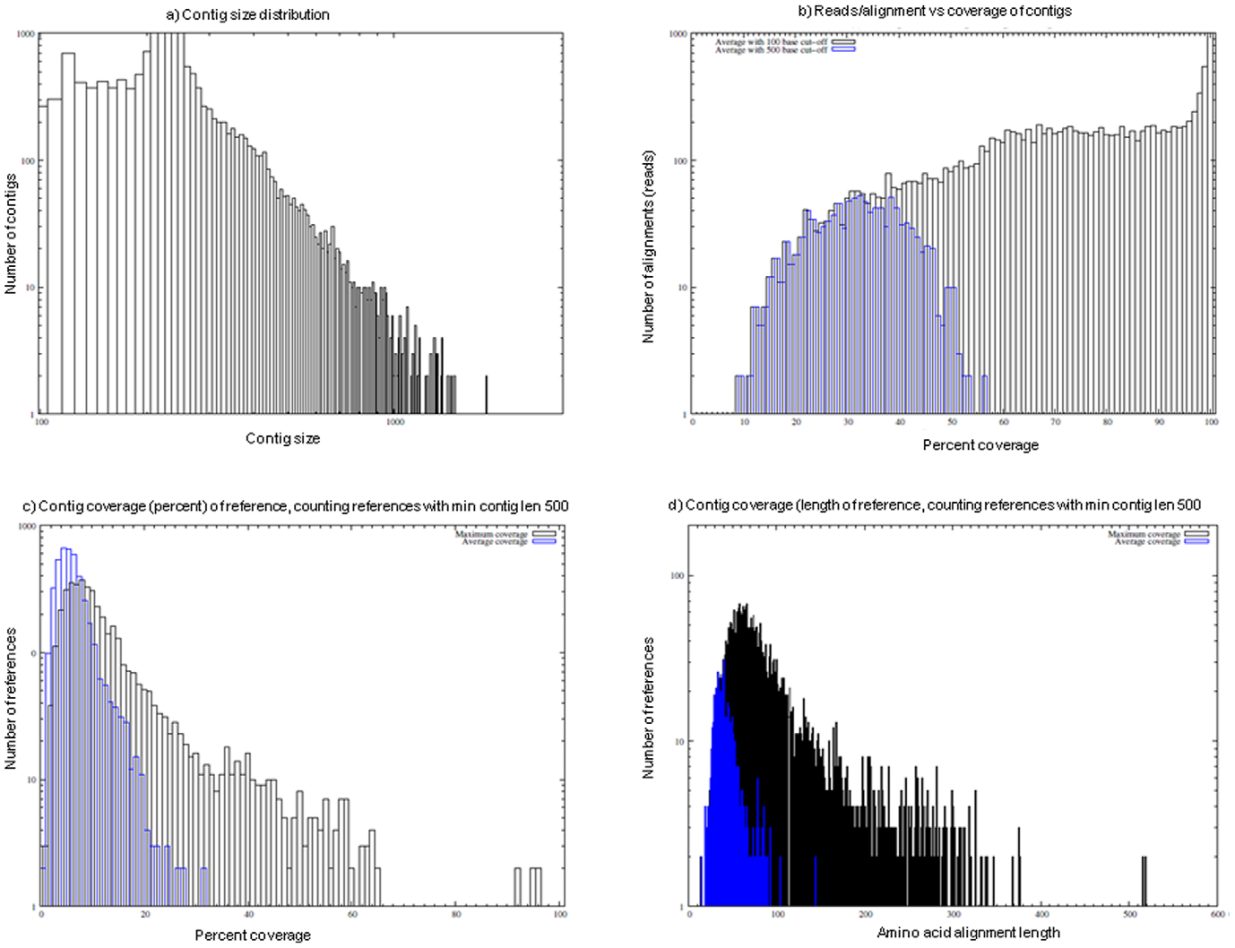
Overview of assembly quality. Contigs were assigned to *An. gambiae* peptide reference using BLASTX with maximum expectation of 10. The cut-off was reduced to realistic levels using the stated minimum alignment lengths. (a) Histogram of contig lengths, smoothed by assigning contigs to partitions, each 10 bp in length. (b) Histogram of read alignment length compared to the contig it was assigned to. Read depth has been calculated from the assembly coordinates of each read, rounded to partitions of 1%. In effect, this shows coverage averaged across all contigs of lengths 100 and 500 bases. 100 bases is inclusive, whereas 500 bases only considers contigs at least twice the length of the original reads. (c) Contig coverage (percentage) of BLAST assigned references, counting references, with minimum contig length of 500 bp. Shown is both the average contig coverage of references, and the maximum coverage, demonstrating some references are covered very well. (d) Repeating Figure c but using amino acid identity length rather than percent identity length.

For most of the reads, the best hits matched a mosquito species in the nr database, most frequently *An. gambiae*. Figure 2 shows the number of best hits for each read matching any of the species represented in the nr database. Key species have been highlighted in Figure 2 with the wide variation in the number of genes in reference species to be noted. Although at a lower frequency, *An. funestus* reads also matched genes from distant species such as human or mouse probably for conserved genes. Analysis of the contig length distribution resulting from the assembly of reads shows a large peak at 240 bp (Figure 3a). The longest contig is 2,226 bp long and made use of 224 reads, coverage varied from 1 to 53 supporting reads.

As expected, the average coverage for short contigs (average with 100 base cut-off) was higher than for longer contigs greater than 500 bases (average with 500 base cut-off) (Figure 3b). Indeed, shorter contigs are more likely to be fully covered by single read which significantly increases their average coverage. In contrary for longer contigs, because there are no reads which fully cover the full length of the contig, their average coverage is lower as shown in Figure 3b.

The coverage based on contig length compared to reference length (*An. gambiae*) is shown in figure 3c. The reference length was normalised to unity and the average (blue) and maximum (black) depth of contigs was counted for each reference sequence. The maximum is effectively the longest contig matching a reference sequence. In each case, the number of reference sequences with the given coverage is summed. This figure shows that some references are very well covered.

The analysis was repeated (Figure 3d) but this time using absolute reference sequence length, showing the contig coverage of some of the longer genes. As can be seen, even the maximum (i.e. most optimistic) measure of reference coverage falters when considering large proteins.

**Figure 4:**
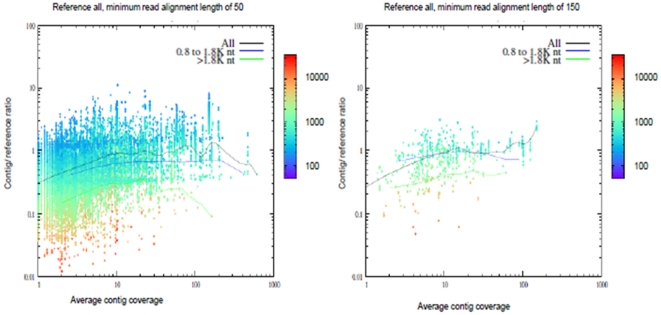
Ratio of contigs and reference length, using TBLASTX against *An. gambiae*, *Ae. aegypti* and *An. funestus*, expectation of E = 10^−3^ and raw score of >45, against average contig coverage (number of reads). Ratios away from unity can be considered hits inside gene families or low scoring false positives, only as the minimum alignment length increases can the validity of the assignment be reasonably certain. Each point is coloured according to reference sequence length, and it can be seen there is a general trend for greater than unity assignments to be to short references and less than unity assignments to longer references. Also included are three lines following three reference cut-off lengths of all, 0.8 to 1.8 K and >1.8 K bp.

The effect of the coverage depth of contigs in the BLASTX analysis is shown in Figure 4 through the ratio of contig and reference lengths. Contigs were compared to *An. gambiae*, *Ae. aegypti* and *An. funestus* using TBLASTX with an expectation of E = 10^−3^ and raw score of >45. Average contig coverage is the average number of reads that make up the individual contigs. Ratios well below unity can be considered hits inside gene families or low scoring false positives, only as the minimum alignment length increases can the validity of the assignment be reasonably certain. Each point is coloured according to reference sequence length, and it can be seen there is a general trend for greater than unity assignments to be to short references and less than unity assignments to longer references. The ratio of the length of individual contigs to the length of their reference orthologue coding region (*An. gambiae*, *Ae. aegypti* and *An. funestus*) increased with the depth of the coverage of the contig as observed previously in another 454 *de novo* assembly [8]. Also included are three lines following three reference cut-off lengths of all, 0.8 to 1.8 K and >1.8 K bp. In all three cases this approaches unity when there are at least 10 contributing reads. It is only the average of all reads that is close to unity. Using the larger minimum read length of 150 bases includes only the high quality alignments, and under these circumstances the three plots have come closer together and become flatter. The remaining contig to-reference assignments are much closer to unity while still being references of approximately 1 kbp or longer.

### Coverage of *An funestus* transcriptome to Gene ontology (GO)

**Figure 5:**
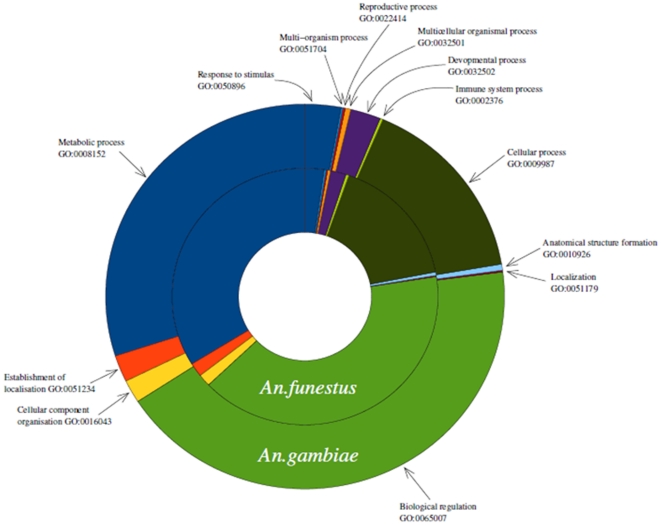
GO pie chart: Pie chart summarizing the distribution of the 23 GO terms covered by *An. funestus* transcriptomes with comparison to *An. gambiae*. These GO terms are one edge away from the root (biological process).

The GO coverage of the *An. funestus* transcriptome 454 contigs was assessed with comparison to that of *An. gambiae* transcriptome reference. For this purpose a Directed Acyclic Graph (DAG) rooted at “biological process” (one of the 3 GO categories with “cellular component” and “molecular function”) was produced for transcriptomes of both species (Figure S1). When “cellular component” was used as the root, this produced a sparsely populated graph for both *An. funestus* and *An. gambiae* transcriptomes, while molecular function produced no graph at all for either species which is different from the GO pattern in *Z. filipendulae* where both “cellular component” and “molecular function” produced significant hits [10]. Sequences from both *An. funestus* 454 contigs and the *An. gambiae* transcriptome reference were assigned to GO reference sequences using BLASTX. The DAG was followed to find all paths from the GO reference sequences to the root. A total of 45,021 paths to the GO category “biological process” were found for *An. funestus* and 88,647 paths for *An. gambiae*. The difference in the number of paths between the two species is probably due to a higher coverage of *An. gambiae* transcriptome than that of *An. funestus*. An analysis of the GO terms found for biological process indicated that *An. funestus* transcriptome as well as the *An. gambiae* reference transcriptome covers 23 terms (Figure S1). The proportion of each of the 23 terms was very similar between these two Anopheles species with the dominant term being “biological regulation” found at 42% in *An. funestus* and 40% in *An. gambiae*. Other major GO terms observed in the graph are “metabolic process” at 29% in *An. funestus* and 33% in *An. gambiae*; “cellular process” at 15% in *An. funestus* and 16% in *An. gambiae*. When moving to a level of two nodes from the root, 197 GO terms were covered by both transcriptomes. Most of these 197 GO terms are associated with only one of the 23 terms (found at one node from root) while few are found in two of the 23 terms (Figure S1). The distribution of these GO terms that were one edge away from the root (“biological process”) is also summarized in a pie chart for both species (Figure 5). The similarity of GO coverage observed between *An. funestus* and *An. gambiae* indicates that the 454 contigs obtained in this study represents a good coverage of *An. funestus* transcriptome. Although most of the GO terms covered by *An. funestus* 454 contigs are general biological terms, some are associated to insect species such as larval behavior, hatching behavior, mating and response to stimulus or stress.

### Transcriptome profiling between resistant and susceptible samples

The most differentially expressed contigs were selected after firstly normalising the reads of each strain to the total reads number. The contigs were then ranked according to the read ratio between resistant and susceptible strains. The most differentially up-regulated contigs in the resistant strain were those with the highest ratio of (r−s)/(r+s) while the top up-regulated in susceptible were those with the highest ratio of (s−r)/(r+s), r and s being the number of resistant and susceptible strain reads in a contig. The analysis of the reads ratio between the resistant strain (FUMOZ-R) and the susceptible strain (Kela) showed a significant differential expression of some ESTs between these samples (Table 2 and Table S1). BLASTX results strongly indicate that the EST (EZ919527) with the highest FUMOZ-R/Kela read ratio (241 reads in FUMOZ-R and zero in Kela) corresponds to a cytochrome P450 gene with the closest match to the *CYP4H18* P450 gene in *An. gambiae* (89% similarity of amino acid sequence). This result correlates with previous findings showing that P450s are involved in pyrethroid resistance in the FUMOZ-R *An. funestus* [11], [12]. It is also located on Chromosome 2 L where a QTL (named *rp_2_*) associated with pyrethroid resistance in the FUMOZ strain was detected [12]. So far, no candidate gene for this *rp_2_* QTL has been identified and *CYP4H18* should be further investigated to assess whether it is associated with *rp_2_* QTL. An EST matching a cuticular protein gene is also highly expressed in the resistant samples suggesting that this enzyme family could potentially be involved in pyrethroid resistance as already been suggested in *An. gambiae* and *An. stephensi* [13], [14].

**Table 2:** Some of the most differentially expressed ESTs (contigs) between FUMOZ and Kela strains.

Contigs and corresponding genes in *An. gambiae*	Accession Number	Number of reads in FUMOZ-R	Number of reads in Kela	Potential function
C4346 (*CYP4H18*) (AGAP002418)	EZ919527	241	0	Cytochrome P450 monooxygenase
C3969 (AGAP004975-PA)	EZ919150	35	0	Prophenoloxidase
C2639 (AGAP000820-PA) (Cuticular gene 125)	EZ917274	19	0	Cuticular protein
C2093 (CYP9J14)	EZ917274	10	0	Cytochrome P450 monooxygenase
C337 (AGAP006485-PA)	EZ915518	0	132	Trypsin putative
C140 (AGAP010182-PA)	EZ915321	1	71	K11802 WD repeat-containing protein 32
C6522 (AGAP012433-PA)	EZ921703	14	210	Calcium channel protein
C3146 (AGAP009805-PH)	EZ918327	1	20	Gustatory receptor

Some ESTs were more highly abundant in the susceptible strain (Table 2). An analysis of these contigs revealed that they belong to genes with a possible role in larval ecology or environmental adaptation. This could be explained by the fact that this is a field strain facing a different ecological environment than that of the laboratory resistant strain. However, this difference could also result from the different geographic origin of the two strains (one from West Africa, Mali and another from Southern Africa in Mozambique). Additionally, the laboratory colonization of the FUMOZ-R strain from Mozambique could have led to significant bottleneck and genetic drift in this strain inducing an artificial selection that may have increased the difference in the transcription profile between the two strains. We cannot also rule out that bias due to incorrect assembly of reads in both strains have not impacted this transcription profile.

### Validation of a set of contigs for gene expression studies by qPCR

qPCR reactions were carried out with a set of six contigs in samples from different life-stages and both sexes used for the transcriptome sequencing (female, male, pupae and 4^th^ instar larvae). These six contigs were chosen mainly because they belong to gene families known to be involved in insecticide resistance such as Glutathione-S-Transferases (*GSTe2* and *GSTd1-3*), P450 genes (*CYP6AH1* and *CYP9J14*) and the cuticular genes 76 and 125.

All the six ESTs were successfully amplified with qPCR products at the expected size in both FUMOZ and Kela strains. They are also expressed in both females and males, in pupae and larvae as well. Although based on a limited number of contigs, this result is an indication that contigs generated from this study could be suitable for gene expression studies in *An. funestus* particularly for microarray study. A custom-designed microarray chip could now be constructed using oligonucleotides generated from the 18,103 contigs of this study. Knowing that no microarray studies using specific *An. funestus* probes have been published so far, this will represent a significant progress for functional genomics in this species.

**Figure 6:**
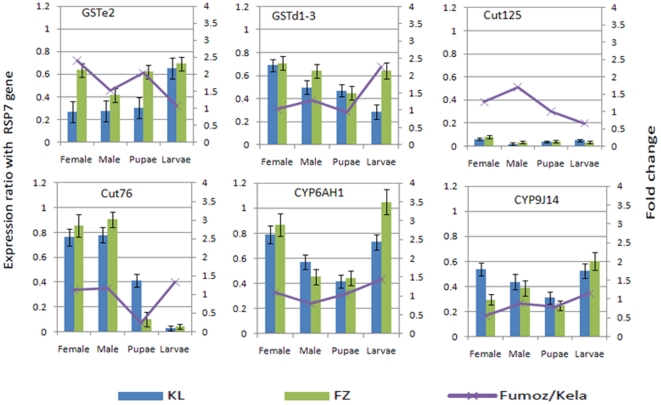
qPCR gene expression profiles of six contigs between the susceptible strain [Kela (field, Mali) and a laboratory pyrethroid-resistant strain (FUMOZ-R). The normalised expression ratio of each contig against the R*SP7* gene is represented on the primary vertical axis while the secondary vertical axis represents FUMOZ-R/Kela fold change of each contig.

The expression levels of the 6 contigs were generally comparable between FUMOZ and Kela [for 20 out of the 24 comparisons (females, males, larvae and pupae for the 6 contigs)]. However, a differential expression between the strains was observed for the contig corresponding to *GSTe2* with a 2.5-fold change (*P*<0.05) for females and 2-fold change in pupae (Figure 6). *GSTe2*, which belongs to the glutathione transferase family, has been found to be involved in detoxification and in insecticide resistance in *An. gambiae*, the other malaria vector [5]. However, although there is a significant difference in expression level for *GSTe2*, between resistant and susceptible, the fold change of the expression is significantly lower than the 18–25 fold change observed for previously identified genes conferring pyrethroid resistance in *An. funestus*, such as the duplicated P450 genes *CYP6P9* and *CYP6P4* [12]. This suggests that if *GSTe2* is playing a role in this resistance, it is probably a minor role. *GSTe2* over-expression was higher in females than in males, a pattern previously observed for other genes related to pyrethroid resistance (*CYP6P9* and *CYP6P4*) in *An. funestus*, where the resistance level is higher in females than in males [12], [15]. A 2.2-fold change over-expression of *GSTd1-3* was also observed in Larvae for FUMOZ compared to Kela while a 4.1-fold change overexpression of the cuticular gene 76 was observed in Kela pupae compared to FUMOZ. The contigs corresponding to the P450s *CYP6AH1* and *CYP9J14* and the cuticular gene 125 did not show a significant difference.

### SNP discovery

The use of two strains in this 454 sequencing, one from the field and the other from the laboratory, offers a good opportunity to identify additional SNPs in *An. funestus*. The MIRA assembler used for the reads assembly identified a total of 31,000 possible SNPs over 4.579 Mb of sequence. To assess the reliability of these SNPs, we carried out a visual inspection of 20 contigs coding for genes from three different biological functions; immunity, detoxification and housekeeping. We used the same criteria as done previously by [8] for SNP calling (at least two occurrence of the minority allele at sites covered by at least five sequences). The results are summarized in Table 3. A total of 14,295 bp of coding sequences was covered revealing 203 SNPs, an average of 1 SNP every 70 bp which is less polymorphic than what was observed for 50 genes previously sequenced using a conventional (SANGER) sequencing method, where 1 SNP was found in every 48 bp [2]. This difference could be due to the fact that genes analysed here were selected from only three biological functions while the previous study was done with a set of genes randomly selected across the genome. This difference could also be due to the more stringent SNP selection used in this study and SNPs of low frequency could have been missed. More SNPs are found as expected in the non coding 3′UTR sequences with 1 SNP every 49 bp but still lower than what was observed with SANGER sequencing [2] of 1SNP every 31 bp for non-coding regions. The 31,000 SNPs are indicated in the contig sequences submitted in TSA by degenerate letters such as Y for C/T, M for A/C etc.

**Table 3:** Nucleotide polymorphism in *An. funestus* genes.

			Coding region											3′UTR				
			Transition				Transversion							Polymorphic sites				
Genes	Accession No	L(bp)	1st	2nd	3rd	Σ	1st	2nd	3rd	Σ	Syn	Rep	Σ	L(bp)	Ts	Tv	Σ	Average bp/SNP
**Immune genes**																		
C3732 as TEP1	EZ918913	647	1	2	7	10	5	1	2	8	10	8	18	/				35.9
C1902as serine protease	EZ917083	947	1	3	9	13	2	0	3	5	11	7	18	54	0	0	0	52.6
C669as trypsin	EZ915850	858	2	3	8	13	2	1	4	7	11	9	20	69	2	1	3	37.3
C1840 as CLIPB3	EZ917021	1019	6	3	6	15	4	3	6	13	11	17	28	152	0	1	2	35.1
C1286 as APL1	EZ916467	792	0	0	4	4	1	0	1	2	5	1	6	294	8	4	12	44
C3582 as glycoprotein	EZ918763	502	0	1	4	5	0	0	4	4	8	1	9	/	0	0	0	55.8
C698 as CLIPB4	EZ915879	532	0	0	0	0	3	0	4	7	0	7	8	285	3	4	7	38
C900 as Toll gene	EZ916081	1012	1	0	8	9	0	1	3	4	11	2	13	188	0	3	3	63.2
C1120 as serpin 3	EZ916301	532	0	2	3	5	0	1	0	1	2	4	6	94	2	1	3	59.1
C2535as serpin 5	EZ917716	494	0	1	1	2	0	1	0	1	2	1	3	194	2	0	2	98.8
C2168as CTLMA1	EZ917349	406	0	0	5	5	2	0	2	4	6	4	10	81	2	2	4	36.9
**Sub-total**		**7741**	**11**	**15**	**55**	**81**	**19**	**8**	**29**	**56**	**77**	**61**	**138**	**1411**	**19**	**16**	**35**	**50.6**
**Detoxification genes**																		
C1920 as ABC transporter	EZ917101	534	0	1	4	5	0	1	0	1	4	2	6	269	6	4	10	33.4
C470 as Heat shock protein 20	EZ915651	980	1	0	7	8	0	1	3	4	10	2	12	329	0	0	0	81.7
C971 as GST e2	EZ916152	462	2	0	3	5	0	0	2	2	5	2	7	86	0	0	0	66
C301 as GSTd1-3	EZ915482	322	1	0	1	2	0	0	0	0	1	1	2	101	1	2	3	64.4
C1414 as CPLC9	EZ916595	806	4	0	4	8	0	1	0	1	4	5	9	124	2	0	2	73.3
C1738 as CYP6AH1	EZ916919	728	0	1	3	4	0	0	1	1	4	1	5	87	3	2	5	72.8
**Sub-total**		**3832**	**8**	**2**	**22**	**32**	**0**	**3**	**6**	**9**	**28**	**13**	**41**	**996**	**12**	**8**	**20**	**65.2**
**Housekeeping genes**																		
C2174as Ribosomal 60S	EZ917355	639	0	2	1	3	0	0	1	1	2	2	4	99	0	3	3	91.3
C187 as Actin	EZ915368	1240	2	2	4	8	0	1	3	4	6	5	11	223	0	1	1	103.3
C2143 as tubulin	EZ917324	843	0	1	5	6	0	0	3	3	8	1	9	154	0	0	0	93.7
**Sub-total**		**2722**	**2**	**5**	**10**	**17**	**0**	**1**	**7**	**8**	**16**	**8**	**24**	**476**	**0**	**4**	**4**	**96.1**
**Total**		**14295**	**21**	**22**	**87**	**130**	**19**	**12**	**42**	**73**	**121**	**82**	**203**	**2883**	**31**	**28**	**59**	
**Average**														160				55.2

**Figure 7:**
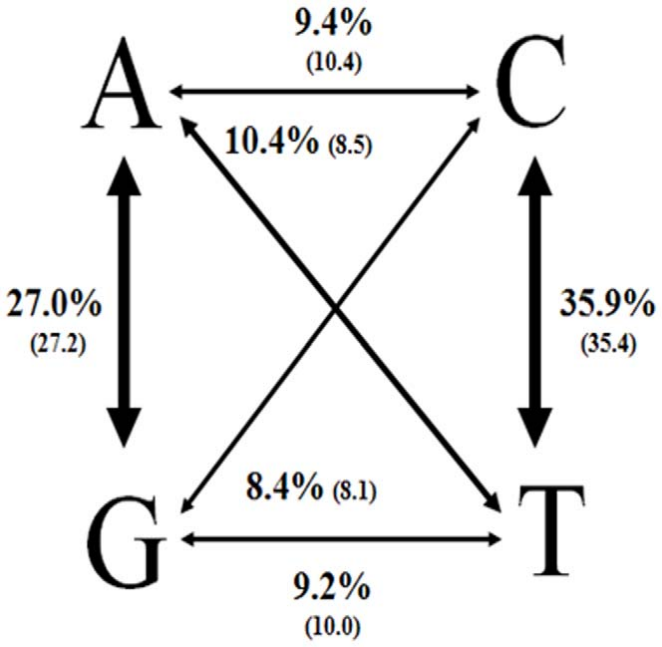
Distribution of transitions and transversions among SNPs. In brackets are the estimates from the SANGER sequencing.

The frequency of transition and transversion substitutions was very similar between the twenty contigs and the previously fifty SANGER sequenced genes (Figure 7). Transition substitutions were more predominant than transversions at the same ratio as previously observed with SANGER sequenced genes (62% vs 38%) [2]. Transitions C↔T and A↔G are over-represented with 35.9 and 27.0% of the total substitutions respectively once again very similar to the previous result (35.4 and 27.2% respectively). The four transversion classes also occurred at similar levels as seen previously (Figure 7). There was a higher frequency of SNPs occurring at the third coding position than in the first and second position as previously observed (Table 3). The difference in the frequency of transversion substitutions previously observed between non-coding regions (3′UTR), coding regions and third coding position was also observed in this analysis (Table 4). These strong correlations suggest that most SNPs identified in this study are likely to be true SNPs. A detailed analysis showed a significant differences in the polymorphism level between genes from the three functional groups, with more polymorphisms observed for immunity genes (1SNP every 51 bp on average) followed by detoxification genes (1SNP every 65 bp) and housekeeping genes (1SNP every 96 bp) (Table 3). This is in accordance with previous studies in *An. funestus* or other mosquitoes indicating a higher genetic variability for genes associated with adaptation such as immunity genes [2], [3], [16]. The average length of 3′UTR in the 20 *An. funestus* contigs was 160 bp compared to 122 bp for *An. gambiae*.

**Table 4:** Transition (T_s_) and transversion (Tv) polymorphisms for different classes of DNA.

	Polymorphism			Probability	
	T_s_	T_v_	%T_v_	Coding Region	3^rd^ coding position
**All genes**					
Non coding regions	31	28	47.4	*P* = 0.021	*P* = 0.01
Coding regions (Cd-R)	130	73	35.9		*P* = 0.204
Third coding position	87	42	32.5		

### Gene conservation between species

The twenty contigs analysed above provided an opportunity to investigate gene conservation between *An. funestus* and related mosquito species such as *An. gambiae*, *Aedes aegypti* and *Culex quinquefasciatus*. Orthologs or similar genes to the *An. funestus* ESTs in the three species were identified by a BLASTX search in vectorbase (http://www.vectorbase.org/index.php) and nr. A ClustalW alignment of all amino acid sequences was carried out and the percentage of similarity estimated. As expected, the highest similarity was always observed between *An. funestus* and *An. gambiae* irrespective of the functional group analysed, with an average of 79.35% similarity between the two anopheline species (Table 5). As reported in previous studies [3], a significant decrease of similarity percentage was observed when compared to *Ae. aegypti* and *Cx quinquefasciatus* (57.75 and 56.5% similarity respectively) which reflects the greater phylogenetic distance between *An. funestus* and these two culicines species. Overall, *An. funestus* ESTs are slightly closer to *Ae. aegypti* than *Cx quinquefasciatus* as also reported previously [3]. This similarity pattern remains the same regardless of functional groups. However, the mean percentage of similarity differs significantly between functional groups with the lowest similarity observed for immune genes, while the housekeeping genes shown the highest similarity between species (Table 5). Genes involved in immunity are known to evolve rapidly, presumably in response to selective pressures imposed by parasites and this could explain both the highest genetic variability and the lowest conservation of these genes in *An. funestus* compared to the other two categories analysed.

**Table 5:** Percentage of similarity between *An. funestus* ESTs and orthologs from three mosquito species.

gene	*An. gambiae*	*Cx quinquefasciatus*	*Ae. aegypti*
**Immune genes**			
C3732 as TEP1	70	40	39
C1902as serine protease	77	55	59
C669as trypsin	66	52	52
C1840 as CLIPB3	62	50	47
C1286 as APL1	63	31	29
C3582 as glycoprotein	90	38	67
C698 as CLIPB4	68	48	53
C900 as Toll gene	88	81	78
C1120 as serpin 3	83	53	48
C2535as serpin 5	74	53	50
C2168as CTLMA1	77	27	28
Average	74.4	48	50
**Detoxification genes**			
C1920 as ABC transporter	77	65	61
C470 as Heat shock protein 20	77	50	56
C971 as GST e2	89	68	66
C301 as GSTd1-3	73	41	36
C1414 as CPLC9	84	47	56
C1738 as CYP6AH1	85	54	62
Average	80.8	54.1	56.1
**Housekeeping genes**			
C2174as Ribosomal 60S	94	89	79
C187 as Actin	96	95	95
C2143 as tubulin	94	93	94
Average	94.7	92.3	89.3
**Total average**	79.35	56.5	57.75

### Conclusion

This study has generated a large set of Expressed Sequence Tags (ESTs) and SNPs that are publicly available for the design of new genomic tools such as microarray or SNP platforms. This will facilitate further functional genomic and genetic studies in this important African malaria vector. In addition it has provided further support for the role of P450 genes in pyrethroid resistant while suggesting that other gene families such as cuticular genes could also be involved.

## Materials and Methods

### Mosquito strains, RNA extraction and synthesis of double-stranded cDNA

Total RNA was extracted from different life-stages of two strains of *An. funestus*, FUMOZ-R a laboratory strain resistant to pyrethroids [15] and a field collected pyrethroid susceptible sample from Kela, a village in Mali (11°88′N, 8°45′W). In order to obtain a large and broad transcriptome data set, RNA was extracted from 10 fourth-instar larvae, 10 pupae, 10 adult females and 10 males from each strain. The use of a field sample was thought likely to increase the genetic diversity of *An. funestus* covered by this study which is particularly useful for SNP discovery.

Total RNA was extracted using the PicoPure RNA isolation kit (Acturus, Mountain View, CA, USA) following the manufacturer's protocol. Total RNA quantity and quality were assessed on a 2% agarose gel and using the Agilent 2100 Bioanalyzer (Agilent technologies, Santa Clara, CA, USA). 10 µl RNA (at around 950 ng/µl) from each of the four life stages were combined to form a RNA pool for each strain. Double stranded cDNA was synthesized from each of these RNA pools using the SMART™ PCR cDNA synthesis kit (Clontech, Mountain View, CA, USA). Briefly, the first strand of the cDNA was synthesized using a mixture of 0.5 µg of total RNA for each pooled sample, 1 µl of 3′SMART CDS primer II A at 12 µM (5′-AAGCAGTGGTATCAACGCAGAGTACT(30)V N-3′), 1 µl of SMART II A oligonucleotide at 12 µM (5′-AAGCAGTGGTATCAACGCAGAGTACGCGGG-3′). After incubation and centrifugation, this mixture was completed with 2 µl of 5× first strand buffer, 1 µl of DTT at 20 µM, 1 µl of dNTPs (10 mM each) and 1 µl of MMLV reverse transcriptase (Clontech, Mountain View, CA, USA). This mixture was incubated for 1 h at 42°C. The double-stranded cDNA was synthesized using the Advantage 2 PCR kit (Clontech, Mountain View, CA, USA) in a 100 µl reaction volume containing 80 µl of distilled water, 10 µl of Advantage 2 PCR buffer, 2 µl of 50× dNTPs (10 mM each), 4 µl of 5′ PCR primer II A at 12 µM (5′-AAGCAGTGGTATCAACGCAGAGT-3′). Amplification was performed with the following conditions: one cycle at 95°C for 1 mn; 18 cycles for 15 sec at 95°C 30 sec at 65°C and 6 min at 68°C.

3 µg of double-stranded cDNA from FUMOZ-R and Kela was sequenced on a 454 GS FLX (Roche, UK) sequencer using the protocol previously described by Margulies [7]. The samples were not normalized to allow the scoring of relative differential gene expression between the susceptible and resistant strains.

### Description of assembly and sequence analysis

Raw 454 reads were trimmed for remaining adapter and poly A/T tails using in-house tools based on a pattern matching algorithm that allowed for base errors. A 6 bp run of Ts within 50 bp of the start is expanded to include all Ts with any number of single base gaps. The read is trimmed from the start to the largest expansion of Ts. Poly As use the same procedure. Adapters are trimmed by matching the largest sub-strings of the known adapter sequence and its complement to the beginning and end of each read. An error of up to 20% was tolerated in the match. The resulting reads were then assembled using Mira (V2.9.15) using the documentation recommended parameters for transcriptome assembly (-AS:nop = 7:rbl = 3 -SK:pr = 80 -AL:mrs = 80 -GE:mxti = yes -454data -454:l454d = yes -CL:msvs = no:qc = no:bsqc = no:pvlc = no:mbc = no:emlc = no -DP:ure = no -OUT:otc = yes). The resulting output files include both gapped and ungapped contigs with quality scores, and an .ace file detailing the exact alignment locations of all reads. Files containing our sequences and their quality scores are available from the National Center for Biotechnology Information (NCBI) Short Read Archive, accession number: SRA009034.

Despite the pooling of different mosquito life stages (with probably different gene expression patterns) in this study, we attempted to identify genes involved in pyrethroid resistance (since differential expression could still be observed) by comparing the transcriptome of the resistant and susceptible strains. With this rationale, we analysed the total reads obtained from the resistant and susceptible samples to identify contigs which showed the most differential expression as these could potentially be associated with pyrethroid resistance. Resistant and susceptible reads were separately assigned by Mira to create contigs, assigned by TBLASTX (E = 10^−3^) to references of *An. gambiae*, *Ae. aegypti* and *An. funestus*. These reads were normalized on the total number of susceptible or resistant reads. Read ratios were compared to the number of normalised contributing reads in each contig.

Quantification of GO term coverage in the GO Directed Acyclic Graph (DAG) was calculated by comparing *An. funestus* 454 contigs to the GO sequence database using BLASTX with E = 10^−5^. To account for the biases inherent in GO, namely over representation of model species, coverage was calculated as a comparison between the absolute coverage of the *An. gambiae* transcriptome (ftp://ftp.vectorbase.org/public_data/organism_data/agambiae/Geneset/agambiae.PEPTIDES-AgamP3.5.fa.gz) and the 454 contigs.

### Quantitative PCR (qPCR) with highly differentially expressed ESTs

We compared the expression pattern of the contigs of the most differentially expressed contigs between the resistant strain FUMOZ-R and the susceptible strain Kela using the GenomeLab GeXP genetic analysis system from Beckman Coulter. Total RNA was extracted from three batches (biological replicates) of fifteen mosquitoes from each life stage (1 day old female and male adults, pupae and fourth-instar larvae) using a PicoPure™ RNA isolation kit (Arkturis) according to manufacturer's instructions. Total RNA quantity and quality were assessed using Nanodrop spectrophotometer (Nanodrop Technologies, Oxfordshire, UK). Primers used are presented in Table 6.

**Table 6:** qPCR primers used.

Genes	Forward/Reverse primers	Product size (bp)
CYP6AH1	*AGGTGACACTATAGAATA*TCGTTTGTGCTTGGGAATGAAATTTGCCTT *GTACGACTCACTATAGGGA*AGTGCAAATCATACTATTAATCGGC	267
CYP9J14	*AGGTGACACTATAGAATA*GTGGCACGTATTTACCGTTTGG GTACGACTCACTATAGGGACACCAACTTGTAGCATTGTTGCG	287
GSTe2	*AGGTGACACTATAGAATA*CTACCATCTCTAGCATTATGGGC GTACGACTCACTATAGGGAGCTTAACCAATTGAATTAATTTC	233
GSTd1-3	*AGGTGACACTATAGAATA*CATTCACCGTTGGTACGAGCGC GTACGACTCACTATAGGGAGCAACACAAAGAAACACGGAGA	208
Cuticular protein 125	*AGGTGACACTATAGAATA*CTTCGGTTAAAGGCGAACTCGCCTG GTACGACTCACTATAGGGACAGCCAGAACCAGTACTCGGCACCG	312
Cuticular protein 76	*AGGTGACACTATAGAATA*CAAGGGCACGGCCACGGAAGGAC GTACGACTCACTATAGGGAGATGGCTTCTGTAAAGCTGTTC	243

The quantitative PCR reaction was carried out using the GenomeLabTM GeXP Start Kit (Beckman and Coulter) according to the protocol provided as described by [12]. The expression level of the *RSP7* ribosomal gene was used to normalise for variation in total cDNA concentration.

## Supporting Information

Figure S1 GO coverage of *An. funestus* 454 contigs compared to *An. gambiae* transcriptome. The GO DAG has no single distance from leaves to root as multiple paths can exist towards the root that are of different lengths. Consequently, depth is based on node to node traversal depth from a root node. All paths from leaf to root were used in the analysis. Node usage was counted by incrementing a node use counter from leaf to root, for all connected nodes between leaf and root, for each blast hit between contig and GO reference sequence. Separate per node counts were maintained for *An. gambiae* and *An. funestus* 454 contigs, making it possible to compare relative node usage and hence relative GO term usage.
(PDF)

Table S1 List of all the contigs successfully annotated by BLASTX against the nr database. Further details are provided such as the number of reads in both strains, read ratio between the two strains, E-values, best hits in *An. gambiae*.
(XLS)
