# Supplementary material for: A De Novo Expression Profiling of Anopheles funestus, Malaria Vector in Africa, Using 454 Pyrosequencing
Source: PLoS One. 2011 Feb 25;6(2):e17418. doi: 10.1371/journal.pone.0017418 (PMC3045460; doi:10.1371/journal.pone.0017418)
Supplement: Figure S1 — GO coverage of An. funestus 454 contigs compared to An. gambiae transcriptome. The GO DAG has no single distance from leaves to root as multiple paths can exist towards the root that are of different lengths. Consequently, depth is based on node to node traversal depth from a root node. All paths from leaf to root were used in the analysis. Node usage was counted by incrementing a node use counter from leaf to root, for all connected nodes between leaf and root, for each blast hit between contig and GO reference sequence. Separate per node counts were maintained for An. gambiae and An. funestus 454 contigs, making it possible to compare relative node usage and hence relative GO term usage. (PDF) [file pone.0017418.s001.pdf]

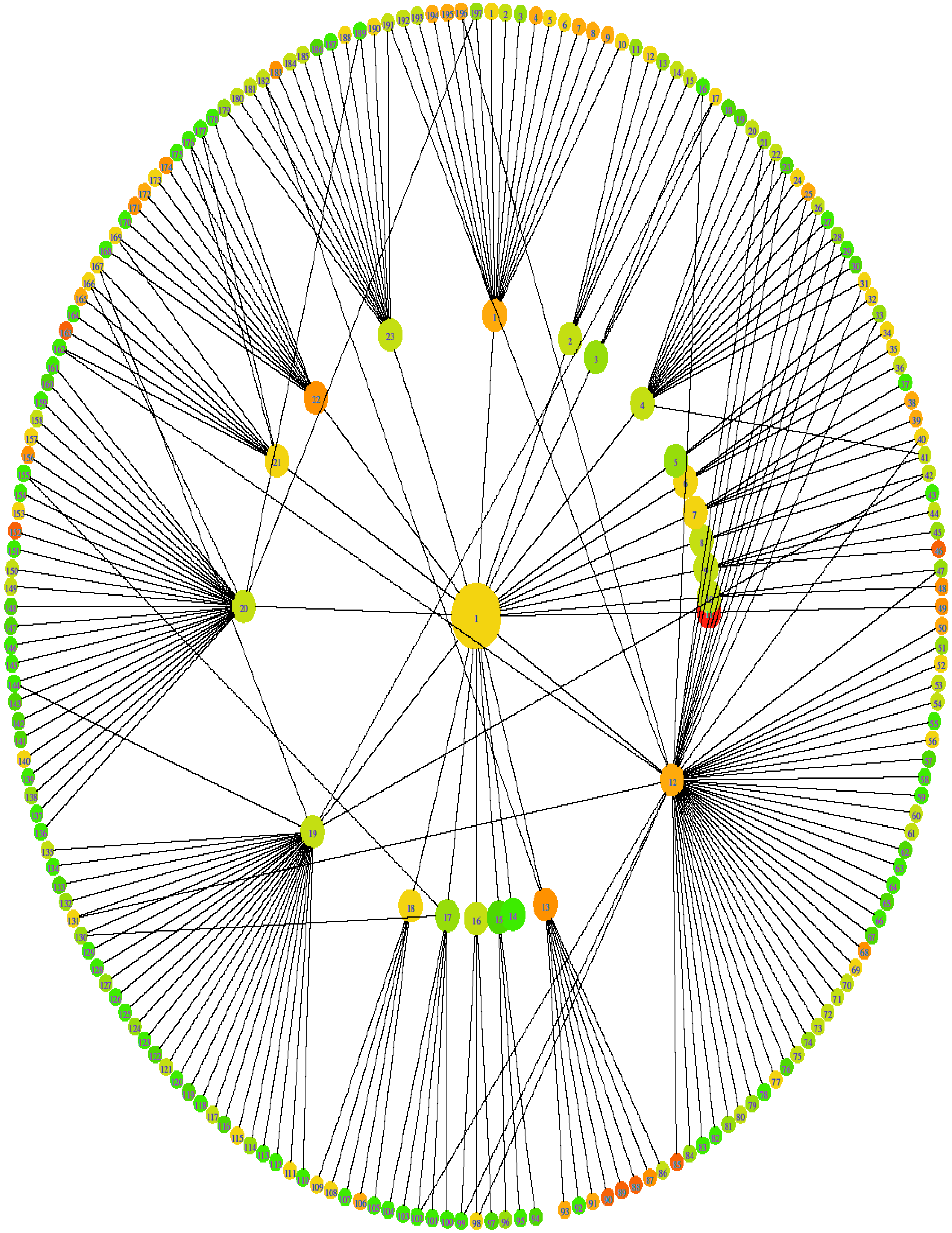

	GO ID	*An. funestus*	*An. gambiae*	GO description
1	GO0008150	45022	88648	biological_process
1	GO0008152	13456	29885	metabolic process
2	GO0051234	1005	1552	establishment of localization
3	GO0043473	10	14	pigmentation
4	GO0016043	869	1345	cellular component organization
5	GO0022610	21	29	biological adhesion
6	GO0065007	19211	35701	biological regulation
7	GO0051179	72	144	localization
8	GO0010926	226	355	anatomical structure formation
9	GO0040011	24	38	locomotion
10	GO0016265	21	31	death
11	GO0001906	1	11	cell killing
12	GO0009987	7135	14658	cellular process
13	GO0002376	107	327	immune system process
14	GO0016032	1	1	viral reproduction
15	GO0000003	12	15	reproduction
16	GO0044085	8	13	cellular component biogenesis
17	GO0040007	55	69	growth
18	GO0048511	29	53	rhythmic process
19	GO0032502	1102	1690	developmental process
20	GO0032501	202	310	multicellular organismal process
21	GO0022414	80	156	reproductive process
22	GO0051704	75	213	multi-organism process
23	GO0050896	1299	2037	response to stimulus
1	GO0018904	13	26	organic ether metabolic process
2	GO0045730	2	3	respiratory burst
3	GO0042445	57	78	hormone metabolic process
4	GO0019637	42	99	organophosphate metabolic process
5	GO0042440	29	52	pigment metabolic process
6	GO0019748	83	150	secondary metabolic process
7	GO0009058	772	1623	biosynthetic process
8	GO0009056	524	1204	catabolic process
9	GO0043170	2267	5095	macromolecule metabolic process
10	GO0006066	261	487	alcohol metabolic process
11	GO0051656	34	45	establishment of organelle localization
12	GO0051236	16	27	establishment of RNA localization
13	GO0051649	190	270	establishment of localization in cell
14	GO0006810	701	1116	transport
15	GO0045184	64	93	establishment of protein localization
16	GO0033059	2	2	cellular pigmentation
17	GO0048066	3	6	pigmentation during development
18	GO0043476	4	5	pigment accumulation
19	GO0043954	4	5	cellular component maintenance
20	GO0043933	225	373	macromolecular complex subunit organization
21	GO0030030	41	55	cell projection organization
22	GO0006996	251	383	organelle organization
23	GO0034330	20	25	cell junction organization
24	GO0022411	33	62	cellular component disassembly
25	GO0045229	6	15	external encapsulating structure organization
26	GO0043062	12	18	extracellular structure organization
27	GO0070193	3	3	synaptonemal complex organization
28	GO0016044	55	78	membrane organization
29	GO0007028	3	3	cytoplasm organization
30	GO0032989	45	56	cellular component morphogenesis
31	GO0006323	4	7	DNA packaging
90	GO0002253	9	33	activation of immune response
91	GO0002252	17	36	immune effector process
92	GO0045058	6	6	T cell selection
93	GO0001776	3	7	leukocyte homeostasis
94	GO0019954	5	6	asexual reproduction
95	GO0019953	3	3	sexual reproduction
96	GO0032505	3	4	reproduction of a single-celled organism
97	GO0042546	4	5	cell wall biogenesis
98	GO0044091	1	2	membrane biogenesis
99	GO0022613	3	3	ribonucleoprotein complex biogenesis
100	GO0030447	4	4	filamentous growth
101	GO0048590	3	3	non-developmental growth
102	GO0016049	15	16	cell growth
103	GO0035265	2	2	organ growth
104	GO0044110	1	1	growth during symbiotic interaction
105	GO0042698	1	1	ovulation cycle
106	GO0022602	7	16	ovulation cycle process
107	GO0007624	1	1	ultradian rhythm
108	GO0007623	10	18	circadian rhythm
109	GO0007622	9	16	rhythmic behavior
110	GO0009838	1	1	abscission
111	GO0010022	1	2	meristem determinacy
112	GO0043934	6	6	sporulation
113	GO0016203	1	1	muscle attachment
114	GO0009791	15	19	post-embryonic development
115	GO0021700	13	25	developmental maturation
116	GO0009790	7	8	embryonic development
117	GO0048856	231	356	anatomical structure development
118	GO0010228	1	1	vegetative to reproductive phase transition
119	GO0019827	6	7	stem cell maintenance
120	GO0001503	4	4	ossification
121	GO0048532	3	5	anatomical structure arrangement
122	GO0007571	4	5	age-dependent general metabolic decline
123	GO0009847	1	1	spore germination
124	GO0007389	104	146	pattern specification process
125	GO0009561	1	1	megagametogenesis
126	GO0030588	2	2	pseudocleavage
127	GO0009653	311	431	anatomical structure morphogenesis
128	GO0031077	1	1	post-embryonic camera-type eye development
129	GO0031076	1	1	embryonic camera-type eye development
130	GO0048589	28	40	developmental growth
131	GO0048869	252	443	cellular developmental process
132	GO0022611	5	7	dormancy process
133	GO0007568	7	8	aging
134	GO0007566	1	1	embryo implantation
135	GO0035188	2	3	hatching
136	GO0035314	1	1	scab formation
137	GO0048316	1	1	seed development
138	GO0007632	3	4	visual behavior
139	GO0043480	1	1	pigment accumulation in tissues
140	GO0048871	3	6	multicellular organismal homeostasis
141	GO0030537	4	5	larval behavior
142	GO0009606	4	5	tropism
143	GO0030534	9	11	adult behavior
144	GO0007275	4	4	multicellular organismal development

32	GO0051825	1	2	adhesion to other organism during symbiotic interaction
33	GO0007155	20	27	cell adhesion
34	GO0050789	18154	33912	regulation of biological process
35	GO0065009	539	937	regulation of molecular function
36	GO0065008	518	852	regulation of biological quality
37	GO0051674	1	1	localization of cell
38	GO0033036	33	71	macromolecule localization
39	GO0031503	2	5	protein complex localization
40	GO0051641	35	66	cellular localization
41	GO0022607	167	261	cellular component assembly
42	GO0048646	59	93	anatomical structure formation involved in morphogenesis
43	GO0031987	1	1	locomotion involved in locomotory behavior
44	GO0033058	2	3	directional locomotion
45	GO0042330	18	25	taxis
46	GO0052192	2	7	movement in environment of other organism during symbiotic interaction
47	GO0008219	19	27	cell death
48	GO0016271	1	3	tissue death
49	GO0001909	1	3	leukocyte mediated cytotoxicity
50	GO0045103	3	7	intermediate filament-based process
51	GO0008037	12	16	cell recognition
52	GO0032940	22	43	secretion by cell
53	GO0022406	3	5	membrane docking
54	GO0022402	101	150	cell cycle process
55	GO0032196	3	3	transposition
56	GO0006413	1	2	translational initiation
57	GO0007059	4	5	chromosome segregation
58	GO0010118	1	1	stomatal movement
59	GO0016037	2	2	absorption of light
60	GO0051651	12	20	maintenance of location in cell
61	GO0007017	57	83	microtubule-based process
62	GO0007163	27	33	establishment or maintenance of cell polarity
63	GO0035212	1	1	cell competition in a multicellular organism
64	GO0007272	9	9	ensheathment of neurons
65	GO0032506	18	22	cytokinetic process
66	GO0009846	1	1	pollen germination
67	GO0030029	51	57	actin filament-based process
68	GO0006276	1	3	plasmid maintenance
69	GO0034621	87	147	cellular macromolecular complex subunit organization
70	GO0007049	7	11	cell cycle
71	GO0019725	62	99	cellular homeostasis
72	GO0003001	9	15	generation of a signal involved in cell-cell signaling
73	GO0008283	25	40	cell proliferation
74	GO0016192	46	65	vesicle-mediated transport
75	GO0070882	9	15	cell wall organization or biogenesis
76	GO0016458	17	19	gene silencing
77	GO0006949	1	2	syncytium formation
78	GO0007302	1	1	nurse cell nucleus anchoring
79	GO0006928	47	67	cell motion
80	GO0007154	222	345	cell communication
81	GO0055085	71	101	transmembrane transport
82	GO0007569	3	3	cell aging
83	GO0006903	4	4	vesicle targeting
84	GO0051301	23	30	cell division
85	GO0001775	40	169	cell activation
86	GO0050900	4	6	leukocyte migration
87	GO0002507	1	3	tolerance induction
88	GO0019882	5	22	antigen processing and presentation
89	GO0045321	35	149	leukocyte activation

145	GO0008340	1	1	determination of adult lifespan
146	GO0009845	1	1	seed germination
147	GO0001763	15	18	morphogenesis of a branching structure
148	GO0048771	4	5	tissue remodeling
149	GO0033555	3	5	multicellular organismal response to stress
150	GO0003008	77	122	system process
151	GO0032898	1	1	neurotrophin production
152	GO0050879	1	4	multicellular organismal movement
153	GO0050878	7	12	regulation of body fluid levels
154	GO0030431	2	2	sleep
155	GO0035264	1	1	multicellular organism growth
156	GO0001816	5	13	cytokine production
157	GO0050817	2	4	coagulation
158	GO0007586	2	3	digestion
159	GO0007585	3	3	respiratory gaseous exchange
160	GO0042303	12	15	molting cycle
161	GO0035187	1	1	hatching behavior
162	GO0048610	9	9	reproductive cellular process
163	GO0019098	6	25	reproductive behavior
164	GO0009566	3	3	fertilization
165	GO0022415	8	18	viral reproductive process
166	GO0048609	25	47	reproductive process in a multicellular organism
167	GO0003006	26	50	reproductive developmental process
168	GO0042710	2	2	biofilm formation
169	GO0007618	1	2	mating
170	GO0048874	2	2	homeostasis of number of cells in a free-living population
171	GO0051707	43	134	response to other organism
172	GO0051705	4	9	behavioral interaction between organisms
173	GO0051703	1	2	intraspecies interaction between organisms
174	GO0044419	17	47	interspecies interaction between organisms
175	GO0000746	2	2	conjugation
176	GO0007567	1	1	parturition
177	GO0007565	1	1	female pregnancy
178	GO0009292	1	1	genetic transfer
179	GO0009628	124	158	response to abiotic stimulus
180	GO0007610	94	148	behavior
181	GO0042221	316	460	response to chemical stimulus
182	GO0006955	27	42	immune response
183	GO0009607	53	168	response to biotic stimulus
184	GO0006950	221	341	response to stress
185	GO0009605	166	260	response to external stimulus
186	GO0009719	70	87	response to endogenous stimulus
187	GO0014823	2	2	response to activity
188	GO0014874	1	2	response to stimulus involved in regulation of muscle adaptation
189	GO0002021	1	1	response to dietary excess
190	GO0051606	73	124	detection of stimulus
191	GO0051716	150	241	cellular response to stimulus
192	GO0015976	2	3	carbon utilization
193	GO0055114	9	15	oxidation reduction
194	GO0006807	1588	3768	nitrogen compound metabolic process
195	GO0044238	2569	5635	primary metabolic process
196	GO0044237	5230	11635	cellular metabolic process
197	GO0044236	7	9	multicellular organismal metabolic process
